# Maturation of beta cells: lessons from in vivo and in vitro models

**DOI:** 10.1007/s00125-022-05672-y

**Published:** 2022-03-04

**Authors:** Tom Barsby, Timo Otonkoski

**Affiliations:** 1 Stem Cells and Metabolism Research Program, Faculty of Medicine, University of Helsinki, Helsinki, Finland; 2 Children’s Hospital, Helsinki University Hospital and University of Helsinki, Helsinki, Finland

**Keywords:** AMPK, Beta cells, Circadian, Differentiation, Islets, Maturation, Metabolism, mTOR, Review, Stem cells

## Abstract

The ability to maintain normoglycaemia, through glucose-sensitive insulin release, is a key aspect of postnatal beta cell function. However, terminally differentiated beta cell identity does not necessarily imply functional maturity. Beta cell maturation is therefore a continuation of beta cell development, albeit a process that occurs postnatally in mammals. Although many important features have been identified in the study of beta cell maturation, as of yet no unified mechanistic model of beta cell functional maturity exists. Here, we review recent findings about the underlying mechanisms of beta cell functional maturation. These findings include systemic hormonal and nutritional triggers that operate through energy-sensing machinery shifts within beta cells, resulting in primed metabolic states that allow for appropriate glucose trafficking and, ultimately, insulin release. We also draw attention to the expansive synergistic nature of these pathways and emphasise that beta cell maturation is dependent on overlapping regulatory and metabolic networks.

**Graphical abstract:**

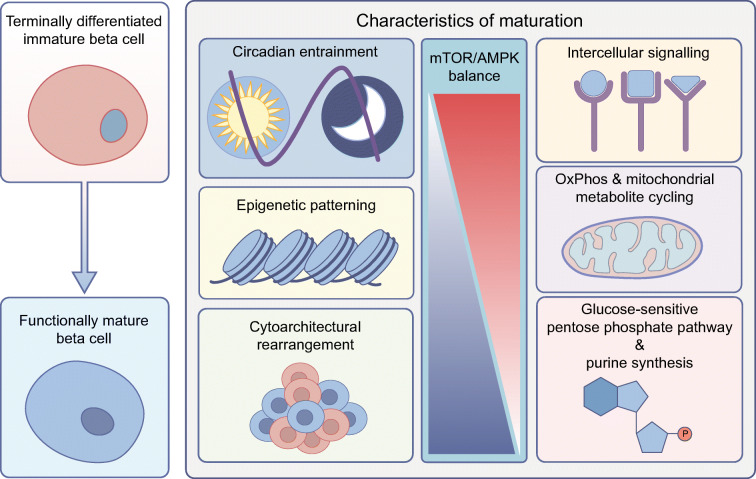

**Supplementary Information:**

The online version contains a slideset of the figures for download, which is available at 10.1007/s00125-022-05672-y.

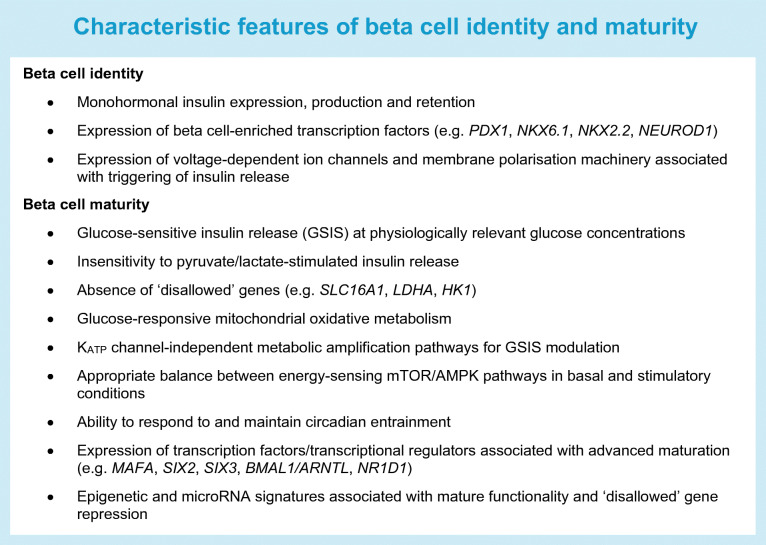



## Introduction

Understanding the mechanisms of beta cell differentiation and maturation is integral to studies of diabetes pathophysiology, regenerative therapies and stem-cell-derived models of beta cell dysfunction and replacement. While the developmental processes that lead to a stable beta cell identity are relatively well known, the mechanisms underlying the functional maturation of beta cells are less clear. In simple terms, beta cell ‘differentiation’ defines the acquisition of a terminally differentiated insulin-positive cell identity throughout in utero development or in vitro stem-cell-based protocols. Conversely, beta cell ‘maturation’ is a measurement of the phenotypic properties of beta cells and their ability to respond to, and control, blood glucose levels through glucose-sensitive insulin secretion (GSIS). Acquisition of this function occurs postnatally in rodents [1–3] and humans [4], and therefore is a property of beta cell development, but one that occurs beyond the attainment of fetal beta cell identity. This divide in cell identity and mature functionality is exemplified in early stem-cell-based differentiation studies that generated beta-like cells with high expression patterns of canonical beta cell markers but with limited functional activity [5, 6]. The issue of defining what constitutes a mature beta cell can be therefore quite problematic if only certain aspects of beta cell biology are investigated, as has been recently reported [7]. Some hallmark features of beta cell ‘identity’ and ‘maturity’ are outlined in the Text box and will be elaborated upon further throughout this review.

The current understanding of beta cell maturation is that such a process is a spectrum rather than a binary state [8–10]. Mature functionality may itself also be a dynamic process, whereby beta cells flux from active to inactive states, or be dependent on the interplay of functionally heterogeneous beta cell pools [11–16]. Functional maturation can be a reversible process, as beta cell dedifferentiation and senescence, with resultant functional deterioration, are known to be associated with the onset of diabetes [17–19]. Therefore, the study of functional maturation of beta cells is necessary to understand the underlying mechanisms of dysfunction eventually leading to diabetes, as well as to improve the efficacy of therapeutic interventions and stem-cell-based islet replacement therapies.

This review will primarily focus on the reported multi-faceted mechanisms that drive and maintain beta cell functional maturation within in vivo and in vitro models.

## Extrinsic triggers and circadian modulation of beta cell maturation

### Nutrient exposure

The functional maturation of mammalian beta cells is known to occur postnatally, and continues to develop post-weaning [1, 2]. There are many potential drivers of this response, the most critical ostensibly being the neonate’s need to adjust to shifting patterns of nutrient consumption and composition. Mouse studies have implicated the change from high amino-acid-based nutrient availability in utero (and the high-fat milk diet of newborns) to pulsatile carbohydrate-based diet post-weaning as a stimulating factor in postnatal beta cell maturation [10, 20, 21]. This change in nutrient type induces a shift in the relative activity of the energy-sensing pathways of mechanistic target of rapamycin (mTOR) complex (mTORC) 1 and AMP-activated protein kinase (AMPK), with functional maturation favouring a basal activity of AMPK signalling. Conversely, the activation of mTORC1 signalling becomes more restricted to periods of glucose stimulation. Intriguingly, the maintenance of a high-fat diet into adulthood retains a more functionally immature beta cell phenotype [21], while the transient inhibition of mTOR in stem-cell-derived islets (SC-islets) improves functional outcomes [20], suggesting that these pathway shifts are causative and not simply incidental to beta cell maturation.

The concept of beneficial mTOR signalling in beta cell differentiation and function is well documented [22]. However, the specific mechanisms driving beta cell maturation through mTOR signalling modulation are still vague. Beta cell-specific overexpression of a kinase-dead mTOR is detrimental to function in mice [23], as is beta cell-specific mTOR knockout [24]. Specific functions of the mTORC1 and -2 complexes have also been implicated in different aspects of beta cell maturation and function. The raptor subunit of the mTORC1 complex is necessary for the regulation of beta cell function, autophagy and repression of disallowed genes [24–26] whereas mTORC2 complexes, which function through the presence of the rictor subunit, have been implicated in the mediation of beta cell mass and proliferation, islet cytoarchitecture and modulation of GSIS through activation of protein kinase Cα [24, 27, 28]. Postnatal rearrangement of islet cytoarchitecture is another key process in mature functionality seen in vivo [29, 30] as well as in recent models of SC-islet maturation in vitro [31]. Modulation of these processes through mTORC2-mediated signalling may indeed be responsible for islet reorganisation, prior to downregulation of mTOR signalling, and the onset of functional maturity.

Another intriguing concept is the coupling of beta cell glucose sensing to mTOR activity. It has recently been shown that acute glucose stimulation of mTORC1 activity in beta cells is only partially dependent on mitochondria-derived glucose metabolism [32]. In line with this, a study reported that glycolytically derived dihydroxyacetone phosphate (DHAP) may signal glucose availability directly to the mTORC1 complex (albeit in human embryonic kidney cells) [33]. Metabolic tracing studies by us and others have shown that SC-islets show strong functional profiles despite limited mitochondrial metabolism of glucose; furthermore, a glycolytic bottleneck beyond the glyceraldehyde-3-phosphate (GA3P)/DHAP enzymatic step is present in SC-islets [34, 35]. It is tempting to speculate that such a direct interplay of glycolytic DHAP generation and mTOR activity may in part be responsible for strong in vitro SC-islet function, without the canonical mitochondrial coupling seen in mature adult islets.

### Circadian clock

In concert with post-weaning feeding cycle and nutrient composition changes, the entrainment of systemic and intrinsic islet circadian clocks has an active role in beta cell maturation in mammals [36–38]. The core circadian clock transcription factors clock circadian regulator (CLOCK) and aryl hydrocarbon receptor nuclear translocator like (BMAL1) are known to cyclically drive the oscillating expression of many beta cell genes necessary for secretory function and regulation of insulin release [39], correlated with the acquisition of GSIS [36]. Although this review focuses primarily on beta cell biology, it is worth noting that the core components of the circadian clock regulate cell-type-specific gene networks within each endocrine population [40]. *BMAL1* (also known as *ARNTL*) and/or *CLOCK* deletion within pancreatic lineages (or beta cells specifically) disrupts the functionality of beta cells, resulting in an oxidative-stress-induced state [41, 42]. Conversely, the overexpression of *Bmal1* was able to increase the amplitude of circadian oscillations and protect against obesity-induced glucose intolerance in mice [43].

**Fig. 1:**
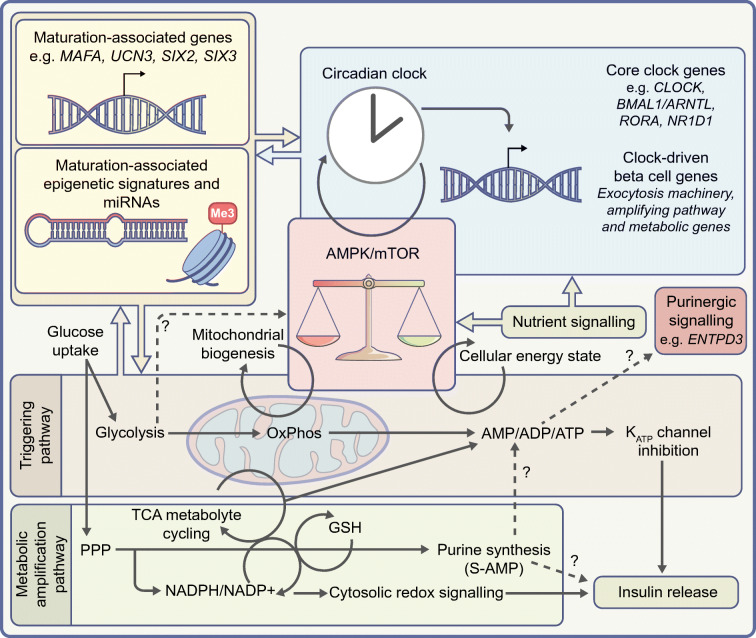
Overlapping transcriptomic, metabolic and energy-sensing machinery that enables the functional maturation of beta cells. The ability of beta cells to derive GSIS is dependent on the synergistic interplay of many metabolic and regulatory features. The post-weaning maturation of beta cells is characterised by the re-balancing of the AMPK/mTOR energy-sensing pathways and their interaction with circadian clock entrainment. Both of these elements further interact with the canonical triggering and metabolic amplification pathways of GSIS involving NADP-mediated glutathione redox cycling. The feedback between these metabolic and nutrient-sensitive control points also trigger/respond to transcriptional shifts of maturation-associated genes, microRNA regulation and epigenetic signatures in beta cells. Dotted arrows with ‘?’ symbols denote indirect or mechanistically unknown pathways of regulation. GSH, glutathione; S-AMP, adenylosuccinate. This figure is available as part of a downloadable slideset

The ability of the circadian clock to rhythmically induce genes that enhance the glucose-sensitive function of mature beta cells may also share some overlap with key components of metabolic energy-sensing pathways [44, 45] (Fig. 1). Indeed, the kinase activity of AMPK is an integral constituent of the clock-cycling mechanism, possibly linking the activity of mTOR/AMPK signalling with clock activity [46–48], and BMAL1 itself is a reported target of the mTOR-effector kinase S6K1 [49]. In a recent study wherein rhythmic circadian clock expression patterns were induced effectively in SC-islets, circadian entrainment as a mechanism for beta cell maturation was shown in principle [50]. Stimulation indices, calcium fluxes and cyclical oxygen consumption were all increased following entrainment. In agreement with this, following SC-islet implantation and maturation in vivo, core clock components *BMAL1*, *RORA* and *BHLHE41* were all upregulated, showing that enhanced beta cell maturation correlates with enhanced expression of core clock components [35]. Perhaps most intriguingly, the recent finding that circadian clock cycling may regulate the alternative splicing of subsets of target genes within beta cells adds a new dimension to the concept of circadian control of beta cell maturation and transcriptional regulation [51].

In aggregate, the triggering of beta cell maturation through the entrainment of circadian clock machinery and the balancing of energy-sensing pathways following postnatal development are key aspects of beta cell functional acquisition. The underlying metabolic shifts that allow for enhanced glucose sensitivity beyond these signals are discussed further below.

## Metabolic control of glucose sensitivity during beta cell maturation

The purpose of this review is not to outline an exhaustive list of all known bioenergetic pathways that couple glucose metabolism to insulin secretion, as many excellent resources already exist for many aspects of beta cell function [52–55]. Instead, our aim is to highlight the interplay between some of these metabolic pathways and their development during beta cell maturation.

### Oxidative phosphorylation

Mitochondrial metabolism, particularly oxidative phosphorylation (OxPhos), is essential for beta cell function [56, 57]. Classically this is encompassed by the ‘triggering pathway’ model of GSIS, whereby mitochondrially generated ATP/ADP ratio shifts inhibit plasma membrane localised K_ATP_ channels, resulting in depolarisation and insulin release [58, 59] (Fig. 1). This acquisition of heightened OxPhos activity during maturation is mirrored in SC-islet studies that demonstrate increased abundance of OxPhos-related genes following enhanced in vitro culture conditions and maturation during murine engraftment [35, 60, 61]. Proteomics, transcriptomics and metabolomics studies within rat islets, as they transition from juvenile-to-adult states, also display enhanced OxPhos gene network signatures [62, 63].

### Tricarboxylic acid cycle-derived metabolites

**Fig. 2:**
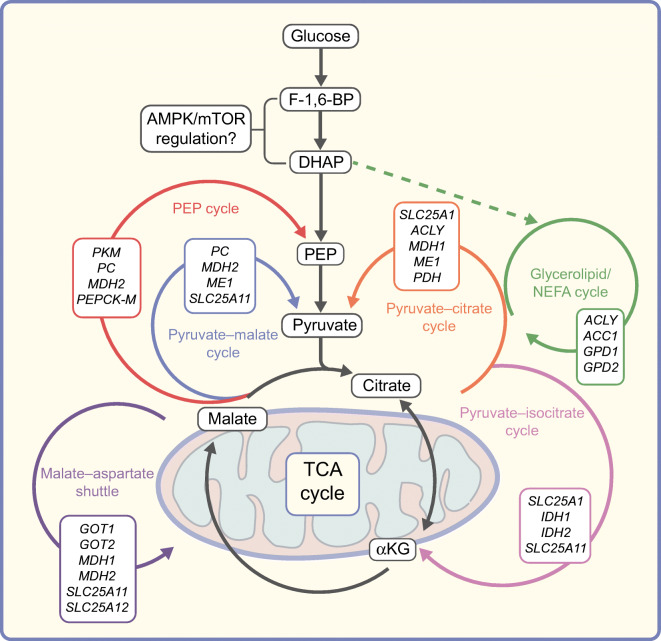
Proposed glucose-sensitive metabolic cycles in functionally mature beta cells. The metabolic processing of glucose into TCA cycle intermediates with the resultant oxidative phosphorylation pathway is a core component of canonical GSIS. However, the processing of TCA-derived metabolites throughout a multitude of mitochondrial–cytosolic cycling reactions have also been shown to be a component of mature beta cell function. Genes that form core components of each cycle are shown in boxes outlined in the colour of the relevant cycle. Glycolytic intermediates may also act in the regulation of glucose-sensitive metabolism, together with interactions with elements of cellular energy-sensing machinery. F-1,6-BP, fructose 1,6-bisphosphate; DHAP, dihydroxyacetone phosphate; PEP, phosphoenolpyruvate; αKG, α-ketoglutarate. This figure is available as part of a downloadable slideset

In parallel with the core OxPhos-mediated triggering pathway model, the cytosolic cycling of numerous mitochondrial metabolites has also been implicated in the generation and maintenance of beta cell function [64, 65]. These proposed metabolite-coupling factors include the malate–aspartate shuttle [66], the pyruvate–malate cycle [67], the pyruvate–citrate cycle [68], the pyruvate–isocitrate cycle [69–71], the phosphoenolpyruvate (PEP) cycle [72, 73] and the glycerolipid/NEFA cycle [74, 75] (Fig. 2). The common thread within most of these cycles is the export and metabolism of tricarboxylic acid (TCA) cycle intermediates that are coupled to GSIS without direct inclusion into OxPhos pathway reactions. However, the extent and importance of each of these cycles in their contribution to beta cell functionality is highly contentious. For instance, within pyruvate–malate and pyruvate–citrate cycling, the activity of the cytosolic malic enzyme (ME1) and the ATP-citrate lyase enzyme (ACLY) are key enzymatic steps. Nevertheless, the genetic reduction of either of these enzymes in beta cell models has been shown to be detrimental to GSIS or to have no detectable effect [67, 68, 76, 77]. A recent proteomic analysis of juvenile-to-adult islet maturation did demonstrate an upregulation in both of these genes, correlating with the acquisition of GSIS functionality [62]. In either case, the unifying concept of malate cycling pathways is the generation of cytosolic NADPH as the coupling factor that augments the insulin release response, ostensibly through the glutathione/redox-mediated modulation of SUMO specific peptidase 1 (SENP1) activity and its interaction with insulin granule release machinery [70] (Fig. 1).

Another TCA-derived pathway, the pyruvate–isocitrate cycle, generates cytosolic NADPH through the activity of the cytosolic isocitrate dehydrogenase enzyme (IDH1). The presence and activity of this enzyme are necessary for beta cell function [69–71]. However, in keeping with the duality of metabolic reports in beta cell models, opposite findings have been reported [78]. It is unlikely that each metabolic cycle acts in isolation. Indeed, many of the TCA metabolite cycles/shuttles rely on overlapping enzymes and mitochondrial carriers (Fig. 2). Mitochondrial citrate export through solute carrier family 25 member 1 (SLC25A1) and α-ketoglutarate import through solute carrier family 25 member 11 (SLC25A11, also known as 2-oxoglutarate carrier [OGC]) are both reliant on malate anti-port. All of which fits with the importance of malate trafficking as a mature metabolic feature of beta cell function. This trafficking may occur through either the pyruvate cycling pathways or the malate–aspartate shuttle function, which ties more directly to OxPhos activity. Indeed, malate import into mitochondria is important for beta cell function (perhaps more important than the cycling kinetics alone) [79]. Appropriate mitochondrial channel expression patterns must therefore be key to glucose-sensitive beta cell function, as repression of either the SLC25A1 or OGC transporters results in the inhibition of GSIS [80, 81]. Intriguingly, direct chemical inhibition of SLC25A1 recapitulates the gene knockdown findings and reduces GSIS [68] but direct inhibition of OGC does not [82].

### Cytosolic redox regulation

Regardless of the relative strengths of each element within these cycles, the core concept relates to that of cytosolic redox regulation through glutathione cycling as a key metabolic coupling factor of glucose metabolism (Fig. 1). Clues relating to this acquisition of glutathione-based redox signalling are seen in human SC-islet models of maturation whereby a multitude of glutathione-related genes, as well as de novo production of glutathione itself, are increased following extended maturation periods in engrafted mice and primary islets [35]. This pattern is also true of aspartate and glutamate, possibly demonstrating an underdeveloped function of malate–aspartate shuttle activity within immature SC-islets as well as limited intracellular glutamate signalling, which has also been implicated as a triggering and amplifying messenger within GSIS [83, 84]. Interestingly, metabolic signatures of the pyruvate–isocitrate cycle are present within SC-islets without extended maturation periods and despite low glucose-responsive OxPhos metabolism [35]. Conversely, the presence of the PEP cycle as a mechanism of GSIS appeared underdeveloped in at least one SC-islet study [34]. It is tempting to conclude, therefore, that a subset of these metabolic cycling pathways may be able to compensate for SC-islet functionality without extended maturation periods, and that each of these pathway cycles may appear independently and throughout beta cell maturation, as is seen in rat islet maturation studies [62, 63, 85].

### Pentose phosphate pathway

The pentose phosphate pathway (PPP) is involved in the coupling of glucose metabolism to insulin release through two complementary mechanisms. The first relates to the cytosolic formation of NADPH from the initial glucose 6-phosphate dehydrogenase (G6PD) and 6-phosphogluconate dehydrogenase (6PGDH) reactions, which may act to fuel the NADPH-dependent cytosolic redox signalling pathway as outlined above [86] (Fig. 1). The second is through the direct formation of adenylosuccinate (S-AMP) and other intermediates within the purine synthesis pathway, downstream from the PPP [87, 88]. The mechanism of coupling of these intermediates to insulin release is still poorly understood, although it is hypothesised that these pre-AMP intermediates may activate AMPK, and therefore modify the mTOR/AMPK energy-sensing axis. This is an intriguing possibility, as the AMP mimetic 5-aminoimidazole-4-carboxamide riboside (AICAR), also an intermediate within the purine synthesis pathway, has been shown to have both positive and negative GSIS modulating properties under acute treatment [89]. AMPK signalling in beta cells has far-reaching implications for many aspects of metabolic network formation and glucose coupling during beta cell maturation [90, 91]. Such signalling may act to modulate the extent of certain metabolic cycling pathways, such as the pyruvate–citrate cycle and glycerolipid/NEFA cycle, through the AMPK-driven inhibition of acetyl-CoA carboxylase 1 (ACC1) [92, 93]. Basal AMPK activation under non-stimulatory glucose concentrations was found to drive the upregulation of mitochondrial OxPhos-related genes in a model of neonatal maturation [21], in line with the concept of enhanced mitochondrial development as an integral factor of mature beta cell functionality. Nevertheless, chronic activation of AMPK has also been shown to have detrimental effects on beta cell functionality [94]. It remains unclear how these glucose-responsive purine pathway intermediates would have beneficial effects on acute GSIS through AMPK activation, if we assume that this activation suppresses mTOR signalling, supposedly the dominant signalling cascade during GSIS [20]. Of course, purine pathway intermediates might not act through AMPK at all; in one metabolic study, an increase in glucose-stimulated 5-aminoimidazole-4-carboxamide ribonucleotide (ZMP) (a phosphorylated form of AICAR) did not result in detectable changes in AMPK activation [88]. Additionally, direct glucose-sensing and reactive oxygen species-sensing capability of AMPK, independent of the cellular energy state (AMP/ADP/ATP ratio), may help to explain this discrepancy and might suggest that glucose-coupled purine synthesis pathways alter GSIS through alternative mechanisms [95, 96].

In summary, the underlying metabolic networks that derive beta cell function are multi-faceted and form progressively throughout beta cell maturation. The specific interplay between energy-sensing machinery, mitochondrial metabolism and metabolite trafficking networks are highly coupled. Therefore, we should be mindful that modifying one aspect of this symphony will have many far-reaching consequences across the biology of the beta cell. However, the generation and stability of these metabolic networks are dependent on the acquisition of appropriate transcriptomic profiles. Candidate pathways and genes that mediate these changes during beta cell maturation are discussed next.

## Signalling pathways and gene markers of beta cell maturation

The extent of beta cell differentiation is generally evaluated, in vitro and in vivo, through the upregulation and maintenance of a set of known beta cell marker genes (including *INS*, *PDX1* [97], *NKX6.1* [98], *NEUROD1* [99], *MAFA* [100] and *UCN3*) [3]. However, the presence of these genes is not necessarily an indication of mature beta cell functionality [10, 101]. Indeed, upregulation of urocortin 3 (UCN3) occurs during the postnatal maturation of beta cells [3, 10] but UCN3 itself appears to be functionally redundant in driving this maturation process [102]. It is therefore important to understand the difference between genes that are critical for maintaining beta cell identity and those that further determine the functional properties of beta cells, and of course, the intrinsic overlap between these two groups. The direct transcriptional regulation of beta cell-specific transcription factors and the influence on metabolic gene regulation is largely unknown. However, certain regulatory patterns have been discovered [103]. For instance, MafA may help repress ‘disallowed’ metabolic genes while maintaining expression of specific glucose transporter genes (*GLUTs*), glucokinase (*GCK*) and *PGC1α* (coding for a regulator of mitochondrial biogenesis and circadian oscillation) within beta cells [104, 105]. A transcriptional network driven by oestrogen-related receptor γ (*ERRγ*) has been shown to regulate multiple OxPhos-related genes, as well as regulating the pyruvate–citrate cycle-related enzyme encoded by *MDH1*, during beta cell maturation [106]. It has also been reported that transcriptional regulation through calcineurin–nuclear factor of activated T cell (NFAT) pathways tailor the expression of *GCK* and *GLUT2* (GLUT2 is the predominant glucose transporter in murine beta cells) [107, 108]. In contrast, the transcription factor activity of regulatory factor X6 (RFX6) is linked to regulation of *GCK* but not *GLUT2* [109]. These overlapping functions in metabolic gene regulation may be due to the web of beta cell-enriched transcription factors directly regulating each other, although there is evidence that the physical interaction of multiple transcription factors is necessary to maintain metabolically mature states, such as the co-binding of neuronal differentiation 1 (NEUROD1) and cAMP responsive element binding protein 1 (CREB1) in beta cell-specific enhancer regions [110].

Transcriptomics studies comparing healthy and diabetic beta cell pools offer many clues to the subsets of genes that may be necessary for maintaining beta cell function, either through candidate transcription factors or through direct regulation of beta cell metabolism. One example of the latter is the higher expression of glucose-6-phosphatase catalytic subunit 2 (*G6PC2*) and 6-phosphofructo-2-kinase/fructose-2,6-biphosphatase 2 (*PFKFB2*) within healthy beta cell samples [111–116] and the upregulation of these genes during maturation of SC-islet beta cells [35, 61]. Both genes encode glycolytically linked enzymes that have been shown to have direct regulatory control over the glucokinase-mediated step of glycolysis [117–120]. The functional maturation of beta cells therefore correlates with heightened control over this initial step of glycolysis, which may regulate the pattern of downstream metabolism and glucose trafficking. The governing mechanisms of glycolytic flux within mature beta cells may also encompass the regulatory effect of cytosolic citrate and PEP (as products of the TCA metabolite cycles outlined previously) on phosphofructokinase 1 (PFK1) activity [112], with the generation of fructose-1,6-bisphosphate as a possible direct modulator of glucose-sensitive AMPK/mTOR activity [95] (Fig. 2). Even the oligomerisation state of the glycolytic enzyme GAPDH (rather than expression level) has been associated with beta cell functional maturation [34]. Some recent findings have also identified the SIX homeobox 2 (SIX2) and SIX homeobox 3 (SIX3) transcription factors as regulators of beta cell functional maturation [121, 122]; this has been demonstrated in SC-islet knockdown models of SIX2, wherein GSIS function was strongly impaired [123]. Interestingly, although SIX2 is necessary for SC-islet functional acquisition in vitro, SIX3 expression appears to be important for advanced maturation events and is not detected in SC-islets in vitro or after extended murine engraftment [35, 123].

The regulation and temporal sequence of genes within this context must at some level be run through transcription factor networks that are responsive to cell lineage signalling and systemic nutritional cues [101]. The ‘holy grail’ within the field of SC-islet generation is an optimised cocktail of signalling and patterning factors that would trigger in vitro beta cell maturation to the same level that is seen post-engraftment. Therefore, SC-islet generation protocols represent fertile ground to test candidate maturation signalling molecules, while simultaneously providing information on processes occurring during postnatal maturation [9, 124, 125].

A recent study found that non-canonical Wnt signalling, through Wnt4, may be one such signalling pathway that triggers maturation events within SC-islet beta cells [126]. It has long been established that Wnt signalling has a variety of important functions throughout islet organogenesis that are spatially and temporally controlled [127]. Furthermore, in SC-islets at earlier stages of differentiation, Wnt signalling affects the balance and penetrance of pancreatic progenitor formation [128, 129]. The exogenous application of Wnt4 to SC-islets increases an assortment of beta cell marker genes as well as mitochondrial OxPhos responsiveness to glucose [126], a pattern that is also seen when Wnt4 is added to human islet and beta cell lines [13]. *WNT4* expression has also been observed in neonatal rat islets, suggesting a role in postnatal functional maturation [85]. However, another SC-islet study was unable to detect any discernible improvement in beta cell maturation following Wnt4 treatment, and conversely found that canonical Wnt signalling inhibition improved SC-islet maturation [130]. Regardless, the interplay of Wnt signalling in different aspects of beta cell differentiation and maturation is well founded. Tantalising evidence of Wnt signalling-derived AMPK/mTOR pathway changes in the regulation of the *Tcf7l2* gene in beta cell proliferation in mice is a clear demonstration of the holistic interactivity of cell signalling, energy-sensing machinery and mature beta cell functionality [131].

Many members of the TGF-β family have also been connected with beta cell maturation, although again with some inconsistent findings between research groups. Inhibition of the transforming growth factor β receptor 1 (TGFBR1, also known as ALK5) during SC-islet maturation has been shown to increase many beta cell marker genes, including *MAFA* [5]. However, more recent studies have shown either a marked improvement of SC-islet beta maturation in the absence of ALK5 inhibitors [132] or, in contrast, an increase in insulin expression in the presence of ALK5 inhibition [31]. Another member of the superfamily, bone morphogenetic protein 4 (BMP4), may also aid in the postnatal maturation of beta cell function following temporally controlled release from islet pericytes [133]. However, one study found that BMP4 treatment inhibited GSIS through the reduction of calcium currents [134], again indicating that particular cellular milieus and developmental timings elicit strong control over specific signalling outcomes. The thyroid hormone triiodothyronine (T3) has also been shown to accelerate the postnatal maturation of beta cells and boost *MAFA* expression in SC-islet models [105, 135], demonstrating that beta cell maturation is affected by systemic hormonal exposure.

In parallel with the signalling pathways outlined above, beta cell maturation may also be self-regulated via the modulation of extracellular ATP release and purinergic receptor-based signalling, through the activity of ectonucleoside triphosphate diphosphohydrolase 3 (ENTPD3) [136]. This has been identified in numerous beta cell transcriptomic studies [114, 137] and has also been shown to be a marker of beta cell maturation within SC-islets [31]. An intriguing overlap between these findings and the model of glucose-sensitive purine synthesis within mature beta cells may imply yet another nexus point of cellular energy state (through AMPK/mTOR modulation), metabolic trafficking (ATP production and release) and the regulation of GSIS in mature beta cells [138] (Fig. 1).

Another intriguing feature of beta cell functional maturation is the regulatory influence of microRNAs. Shifting patterns of microRNA expression have been shown to elicit robust regulatory effects on metabolic gene expression and beta cell functionality in a nutrient-sensitive manner, as well as throughout postnatal maturation [139–141]. The upregulation of the miR-129 family in beta cells during postnatal weaning in mice correlated with enhanced glucose-responsive insulin release [139]. This mirrors the postnatal increase in the expression of the miR-29 family, which has also been shown to repress the ‘disallowed’ genes *REST* [139] and *SLC16A1* [142]. In contrast, downregulation of the miR-181 and miR-17 families during postnatal maturation leads to the upregulation of *GPD2*, *MDH1* and *PFKP* metabolic genes [139]. Other microRNAs such as the miR-223 family (which ostensibly maintains *PDX1* and *NKX6.1* expression through suppression of forkhead box O1 [FOXO1] and SRY-box transcription factor 6 [SOX6] pathways [143]) and the miR-7 family (which reportedly boosts GSIS and *PDX1* levels in SC-islets [144] while suppressing mTOR signalling and proliferation [145]) are all enriched in mature beta cells. However, conclusions about the presence or absence of a particular microRNA family should be assessed in relative terms. For example, the miR-375 family is upregulated during SC-islet maturation [144], yet the forced overexpression of miR-375 in primary islets was reported to blunt GSIS responses and reduce glucose-responsive OxPhos [146]. This drop in functional activity could be explained by the increased expression of *PDK4* and reduced *PC* and *MDH1* expression within the primary islets. The shifting patterns and balance of microRNA family expression is therefore another key component of the onset and maintenance of beta cell maturity.

Finally, epigenetic signatures may help explain particular functional features of mature and immature beta cells. Both DNA methylation and histone modification are mechanisms by which beta cell identity and function are maintained, through tailoring the expression pattern of beta cell-enriched transcription factors, as well as being regulated by the transcription factors themselves [147, 148]. Some relevant examples include DNA methylation through the activity of DNA methyltransferase 3 α (DNMT3A), which has been linked to the repression of beta cell ‘disallowed’ genes, regulated through the mTORC1 component raptor [26] and through the inhibition of Wnt signalling during SC-islet maturation [130]. Histone methylation involving the activity of the polycomb repressor complex (PRC2) may act in juvenile islets to maintain an immature transcriptomic state together with trithorax group (TrxG) proteins [121, 149]. Evidence for extensive epigenetic shifts throughout beta cell maturation has also been seen in SC-islets [50]. All of these aspects of regulation are intricately tied to the metabolic state of the beta cell, as each form of epigenetic modification is fuelled by specific metabolic inputs [150].

In summary, the concept of tracking beta cell maturation through panels of marker genes is one that should be approached cautiously. While core beta cell identity genes are no doubt important for many facets of beta cell maturation, the upregulation of one particular gene is not necessarily a strong argument for predicting functional maturation. Indeed, much more needs to be uncovered about how signalling pathways fully trigger beta cell maturity and through which mechanisms they operate. Furthermore, interpreting expression levels of particular genes, especially those within core metabolic pathways, should be done carefully so as not to misconstrue what is necessary for specific beta cell function and what is key for basal cellular metabolism. Additionally, overexpression of particular genes within a pathway may not necessarily trigger systemic maturation events. Full understanding of beta cell maturity clearly needs to go beyond simplistic models of gene and protein expression, and the regulation and maintenance of epigenetic signatures in beta cell function and disease must be considered.

## Concluding remarks

Beta cell maturation is a multi-faceted process that takes cues from systemic nutritional and hormonal signals and ultimately results in a primed transcriptomic and metabolic beta cell state conducive to drive GSIS (Fig. 1). Recent advances have uncovered many of the core factors and machinery that are necessary to achieve functional maturity, and have woven together how these gene and metabolic networks form and maintain beta cell function. However, a unified model of the acquisition of beta cell functional maturation has yet to be completed. Even so, the vast interconnectedness and synergistic properties of cellular energy-sensing, signalling pathways, metabolic networks and transcriptional regulation in the generation of this maturation state is clear. We hope that this review has highlighted how each element of reported beta cell function is intricately aligned with many other aspects of beta cell biology, and that phenotypic outcomes of gene-knockout or chemical-intervention studies may elicit robust changes beyond the expression patterns of canonical beta cell markers. The recent application of sequencing, metabolic tracing and proteomic assays to probe beta cell maturation and dysfunction offers an incredible resource with which to better understand the acquisition of beta cell functionality, ultimately aiding in the understanding of diabetic pathologies and in the development of novel therapies.

## Supplementary information

ESM 1 (PPTX 332 kb)

## Abbreviations

AICAR: 5-Aminoimidazole-4-carboxamide riboside
ALK5: Transforming growth factor β receptor 1
AMPK: AMP-activated protein kinase
BMAL1: Aryl hydrocarbon receptor nuclear translocator like
BMP4: Bone morphogenetic protein 4
DHAP: Dihydroxyacetone phosphate
GSIS: Glucose-sensitive insulin release
mTOR: Mechanistic target of rapamycin
mTORC: mTOR complex
OGC: Solute carrier family 25 member 11
OxPhos: Oxidative phosphorylation
PEP: Phosphoenolpyruvate
PPP: Pentose phosphate pathway
SC-islets: Stem-cell-derived islets
SIX2: SIX homeobox 2
SIX3: SIX homeobox 3
SLC25A1: Solute carrier family 25 member 1
TCA: Tricarboxylic acid
UCN3: Urocortin 3

## References

[1] Bliss CR, Sharp GWG (1992). Glucose-induced insulin release in islets of young rats: time-dependent potentiation and effects of 2-bromostearate. Am J Physiol Endocrinol Metab.

[2] Stolovich-Rain M, Enk J, Vikesa J (2015). Weaning triggers a maturation step of pancreatic β cells. Dev Cell.

[3] Blum B, Hrvatin S, Schuetz C, Bonal C, Rezania A, Melton DA (2012). Functional beta-cell maturation is marked by an increased glucose threshold and by expression of urocortin 3. Nat Biotechnol.

[4] Otonkoski T, Andersson S, Knip M, Simell O (1988). Maturation of insulin response to glucose during human fetal and neonatal development. Studies with perifusion of pancreatic isletlike cell clusters. Diabetes.

[5] Rezania A, Bruin JE, Arora P (2014). Reversal of diabetes with insulin-producing cells derived in vitro from human pluripotent stem cells. Nat Biotechnol.

[6] Pagliuca FW, Millman JR, Gürtler M (2014). Generation of functional human pancreatic β cells in vitro. Cell.

[7] Kaestner KH, Campbell-Thompson M, Dor Y (2021). What is a β cell? - chapter I in the human islet research network (HIRN) review series. Mol Metab.

[8] Veres A, Faust AL, Bushnell HL (2019). Charting cellular identity during human in vitro β-cell differentiation. Nature.

[9] Velazco-Cruz L, Goedegebuure MM, Millman JR (2020). Advances toward engineering functionally mature human pluripotent stem cell-derived β cells. Front Bioeng Biotechnol.

[10] Zeng C, Mulas F, Sui Y (2017). Pseudotemporal ordering of single cells reveals metabolic control of postnatal β cell proliferation. Cell Metab.

[11] Pipeleers DG (1992). Heterogeneity in pancreatic β-cell population. Diabetes.

[12] Benninger RKP, Hodson DJ (2018). New understanding of β-cell heterogeneity and in situ islet function. Diabetes.

[13] Bader E, Migliorini A, Gegg M (2016). Identification of proliferative and mature β-cells in the islets of langerhans. Nature.

[14] Yang C, Galivo F, Dorrell C (2017). Is a β cell a β cell?. Curr Opin Endocrinol Diabetes Obes.

[15] Avrahami D, Klochendler A, Dor Y, Glaser B (2017). Beta cell heterogeneity: an evolving concept. Diabetologia.

[16] Farack L, Golan M, Egozi A (2019). Transcriptional heterogeneity of Beta cells in the intact pancreas. Dev Cell.

[17] Weir GC, Bonner-Weir S (2004). Five of stages of evolving β-cell dysfunction during progression to diabetes. Diabetes.

[18] Thompson PJ, Shah A, Ntranos V, Van Gool F, Atkinson M, Bhushan A (2019). Targeted elimination of senescent Beta cells prevents type 1 diabetes. Cell Metab.

[19] Salinno C, Cota P, Bastidas-Ponce A, Tarquis-Medina M, Lickert H, Bakhti M (2019). β-Cell maturation and identity in health and disease. Int J Mol Sci.

[20] Helman A, Cangelosi AL, Davis JC (2020). A nutrient-sensing transition at birth triggers glucose-responsive insulin secretion. Cell Metab.

[21] Jaafar R, Tran S, Shah AN (2019). mTORC1-to-AMPK switching underlies β cell metabolic plasticity during maturation and diabetes. J Clin Invest.

[22] Ardestani A, Lupse B, Kido Y, Leibowitz G, Maedler K (2018). mTORC1 signaling: a double-edged sword in diabetic β cells. Cell Metab.

[23] Alejandro EU, Bozadjieva N, Blandino-Rosano M (2017). Overexpression of kinase-dead mTOR impairs glucose homeostasis by regulating insulin secretion and not β-cell mass. Diabetes.

[24] Sinagoga KL, Stone WJ, Schiesser JV (2017). Distinct roles for the mTOR pathway in postnatal morphogenesis, maturation and function of pancreatic islets. Development.

[25] Blandino-Rosano M, Barbaresso R, Jimenez-Palomares M (2017). Loss of mTORC1 signalling impairs β-cell homeostasis and insulin processing. Nat Commun.

[26] Ni Q, Gu Y, Xie Y (2017). Raptor regulates functional maturation of murine beta cells. Nat Commun.

[27] Xie Y, Cui C, Nie A (2017). The mTORC2/PKC pathway sustains compensatory insulin secretion of pancreatic β cells in response to metabolic stress. Biochim Biophys Acta, Gen Subj.

[28] Yuan T, Lupse B, Maedler K, Ardestani A (2018). mTORC2 signaling: a path for pancreatic β Cell’s growth and function. J Mol Biol.

[29] Adams MT, Gilbert JM, Hinojosa Paiz J, Bowman FM, Blum B (2018). Endocrine cell type sorting and mature architecture in the islets of Langerhans require expression of roundabout receptors in β cells. Sci Rep.

[30] Aguayo-Mazzucato C, Sanchez-Soto C, Godinez-Puig V, Gutiérrez-Ospina G, Hiriart M (2006). Restructuring of pancreatic islets and insulin secretion in a postnatal critical window. PLoS One.

[31] Docherty FM, Riemondy KA, Castro-Gutierrez R (2021). ENTPD3 Marks mature stem cell-derived β-cells formed by self-aggregation in vitro. Diabetes.

[32] Rumala CZ, Liu J, Locasale JW, Corkey BE, Deeney JT, Rameh LE (2020). Exposure of pancreatic β-cells to excess glucose results in bimodal activation of mTORC1 and mTOR-dependent metabolic acceleration. iScience.

[33] Orozco JM, Krawczyk PA, Scaria SM (2020). Dihydroxyacetone phosphate signals glucose availability to mTORC1. Nat Metab.

[34] Davis JC, Alves TC, Helman A (2020). Glucose response by stem cell-derived β cells in vitro is inhibited by a bottleneck in glycolysis. Cell Rep.

[35] Balboa D, Barsby T, Lithovius V et al (2022) Functional, metabolic and transcriptional maturation of human pancreatic islets derived from stem cells. Nat Biotechnol In Press 10.1038/s41587-022-01219-z PMC9287162 35241836

[36] Rakshit K, Qian J, Satish Gaonkar K (2018). Postnatal ontogenesis of the islet circadian clock plays a contributory role in β-cell maturation process. Diabetes.

[37] Vieira E, Burris TP, Quesada I (2014). Clock genes, pancreatic function, and diabetes. Trends Mol Med.

[38] Pulimeno P, Mannic T, Sage D (2013). Autonomous and self-sustained circadian oscillators displayed in human islet cells. Diabetologia.

[39] Perelis M, Marcheva B, Ramsey KM et al (2015) Pancreatic β cell enhancers regulate rhythmic transcription of genes controlling insulin secretion. Science 350(6261):aac4250. 10.1126/science.aac4250 10.1126/science.aac4250 PMC4669216 26542580

[40] Petrenko V, Saini C, Giovannoni L (2017). Pancreatic α- and β-cellular clocks have distinct molecular properties and impact on islet hormone secretion and gene expression. Genes Dev.

[41] Marcheva B, Ramsey KM, Buhr ED (2010). Disruption of the CLOCK components CLOCK and BMAL1 leads to hypoinsulinaemia and diabetes. Nature.

[42] Lee J, Moulik M, Fang Z (2013). Bmal1 and β-cell clock are required for adaptation to circadian disruption, and their loss of function leads to oxidative stress-induced β-cell failure in mice. Mol Cell Biol.

[43] Rakshit K, Matveyenko AV (2021). Induction of Core circadian clock transcription factor Bmal1 enhances β-cell function and protects against obesity-induced glucose intolerance. Diabetes.

[44] Bass J, Takahashi JS (2010). Circadian integration of metabolism and energetics. Science.

[45] Perelis M, Ramsey KM, Bass J (2015). The molecular clock as a metabolic rheostat. Diabetes Obes Metab.

[46] Jordan SD, Lamia KA (2013). AMPK at the crossroads of circadian clocks and metabolism. Mol Cell Endocrinol.

[47] Lee Y, Kim EK (2013). AMP-activated protein kinase as a key molecular link between metabolism and clockwork. Exp Mol Med.

[48] Sato F, Kohsaka A, Bhawal UK, Muragaki Y (2018). Potential roles of dec and bmal1 genes in interconnecting circadian clock and energy metabolism. Int J Mol Sci.

[49] Lipton JO, Yuan ED, Boyle LM (2015). The circadian protein BMAL1 regulates translation in response to S6K1-mediated phosphorylation. Cell.

[50] Alvarez-Dominguez JR, Donaghey J, Rasouli N (2020). Circadian entrainment triggers maturation of human in vitro islets. Cell Stem Cell.

[51] Marcheva B, Perelis M, Weidemann BJ (2020). A role for alternative splicing in circadian control of exocytosis and glucose homeostasis. Genes Dev.

[52] Nicholls DG (2016). The pancreatic β-cell: a bioenergetic perspective. Physiol Rev.

[53] Kalwat MA, Cobb MH (2017). Mechanisms of the amplifying pathway of insulin secretion in the β cell. Pharmacol Ther.

[54] Spégel P, Mulder H (2020). Metabolomics analysis of nutrient metabolism in β-cells. J Mol Biol.

[55] Campbell JE, Newgard CB (2021). Mechanisms controlling pancreatic islet cell function in insulin secretion. Nat Rev Mol Cell Biol.

[56] Maechler P (2013). Mitochondrial function and insulin secretion. Mol Cell Endocrinol.

[57] Malmgren S, Nicholls DG, Taneera J (2009). Tight coupling between glucose and mitochondrial metabolism in clonal beta-cells is required for robust insulin secretion. J Biol Chem.

[58] Ashcroft FM, Harrison DE, Ashcroft SJ (1984). Glucose induces closure of single potassium channels in isolated rat pancreatic beta-cells. Nature.

[59] Cook DL, Hales N (1984). Intracellular ATP directly blocks K+ channels in pancreatic B-cells. Nature.

[60] Nair GG, Liu JS, Russ HA (2019). Recapitulating endocrine cell clustering in culture promotes maturation of human stem-cell-derived β cells. Nat Cell Biol.

[61] Augsornworawat P, Maxwell KG, Velazco-Cruz L, Millman JR (2020). Single-cell transcriptome profiling reveals β cell maturation in stem cell-derived islets after transplantation. Cell Rep.

[62] Wortham M, Benthuysen JR, Wallace M (2018). Integrated in vivo quantitative proteomics and nutrient tracing reveals age-related metabolic rewiring of pancreatic β cell function. Cell Rep.

[63] Jermendy A, Toschi E, Aye T (2011). Rat neonatal beta cells lack the specialised metabolic phenotype of mature beta cells. Diabetologia.

[64] Jensen MV, Joseph JW, Ronnebaum SM, Burgess SC, Sherry AD, Newgard CB (2008). Metabolic cycling in control of glucose-stimulated insulin secretion. Am J Physiol Endocrinol Metab.

[65] Ferdaoussi M, MacDonald PE (2017). Toward connecting metabolism to the Exocytotic site. Trends Cell Biol.

[66] Eto K, Suga S, Wakui M (1999). NADH shuttle system regulates K (ATP) channel-dependent pathway and steps distal to cytosolic Ca2+concentration elevation in glucose-induced insulin secretion. J Biol Chem.

[67] Pongratz RL, Kibbey RG, Shulman GI, Cline GW (2007). Cytosolic and mitochondrial malic enzyme isoforms differentially control insulin secretion. J Biol Chem.

[68] Guay C, Madiraju SRM, Aumais A, Joly É, Prentki M (2007). A role for ATP-citrate lyase, malic enzyme, and pyruvate/citrate cycling in glucose-induced insulin secretion. J Biol Chem.

[69] Ronnebaum SM, Ilkayeva O, Burgess SC (2006). A pyruvate cycling pathway involving cytosolic NADP-dependent isocitrate dehydrogenase regulates glucose-stimulated insulin secretion. J Biol Chem.

[70] Ferdaoussi M, Dai X, Jensen MV (2015). Isocitrate-to-SENP1 signaling amplifies insulin secretion and rescues dysfunctional β cells. J Clin Invest.

[71] Zhang G-F, Jensen MV, Gray SM (2021). Reductive TCA cycle metabolism fuels glutamine- and glucose-stimulated insulin secretion. Cell Metab.

[72] Stark R, Pasquel F, Turcu A (2009). Phosphoenolpyruvate cycling via mitochondrial phosphoenolpyruvate carboxykinase links anaplerosis and mitochondrial GTP with insulin secretion. J Biol Chem.

[73] Abulizi A, Cardone RL, Stark R (2020). Multi-tissue acceleration of the mitochondrial phosphoenolpyruvate cycle improves whole-body metabolic health. Cell Metab.

[74] Nolan CJ, Madiraju MSR, Delghingaro-Augusto V, Peyot ML, Prentki M (2006). Fatty acid signaling in the β-cell and insulin secretion. Diabetes.

[75] Prentki M, Corkey BE, Madiraju SRM (2020). Lipid-associated metabolic signalling networks in pancreatic beta cell function. Diabetologia.

[76] Ronnebaum SM, Jensen MV, Hohmeier HE (2008). Silencing of cytosolic or mitochondrial isoforms of malic enzyme has no effect on glucose-stimulated insulin secretion from rodent islets. J Biol Chem.

[77] Joseph JW, Odegaard ML, Ronnebaum SM (2007). Normal flux through ATP-citrate lyase or fatty acid synthase is not required for glucose-stimulated insulin secretion. J Biol Chem.

[78] Guay C, Joly E, Pepin E (2013). A role for cytosolic isocitrate dehydrogenase as a negative regulator of glucose signaling for insulin secretion in pancreatic ß-cells. PLoS One.

[79] Heart E, Cline GW, Collis LP, Pongratz RL, Gray JP, Smith PJS (2009). Role for malic enzyme, pyruvate carboxylation, and mitochondrial malate import in glucose-stimulated insulin secretion. Am J Physiol Endocrinol Metab.

[80] Joseph JW, Jensen MV, Ilkayeva O (2006). The mitochondrial citrate/isocitrate carrier plays a regulatory role in glucose-stimulated insulin secretion. J Biol Chem.

[81] Odegaard ML, Joseph JW, Jensen MV (2010). The mitochondrial 2-oxoglutarate carrier is part of a metabolic pathway that mediates glucose- and glutamine-stimulated insulin secretion. J Biol Chem.

[82] Stamenkovic JA, Andersson LE, Adriaenssens AE (2015). Inhibition of the malate-aspartate shuttle in mouse pancreatic islets abolishes glucagon secretion without affecting insulin secretion. Biochem J.

[83] Maechler P (2017). Glutamate pathways of the beta-cell and the control of insulin secretion. Diabetes Res Clin Pract.

[84] Gheni G, Ogura M, Iwasaki M (2014). Glutamate acts as a key signal linking glucose metabolism to incretin/cAMP action to amplify insulin secretion. Cell Rep.

[85] Martens GA, Motté E, Kramer G (2014). Functional characteristics of neonatal rat β cells with distinct markers. J Mol Endocrinol.

[86] Spégel P, Sharoyko VV, Goehring I (2013). Time-resolved metabolomics analysis of β-cells implicates the pentose phosphate pathway in the control of insulin release. Biochem J.

[87] Gooding JR, Jensen MV, Dai X (2015). Adenylosuccinate is an insulin Secretagogue derived from glucose-induced purine metabolism. Cell Rep.

[88] Lorenz MA, El Azzouny MA, Kennedy RT, Burant CF (2013). Metabolome response to glucose in the β-cell line INS-1 832/13. J Biol Chem.

[89] Fu A, Eberhard CE, Screaton RA (2013). Role of AMPK in pancreatic beta cell function. Mol Cell Endocrinol.

[90] Rourke JL, Hu Q, Screaton RA (2017). AMPK and friends: central regulators of β cell biology. Trends Endocrinol Metab.

[91] Szkudelski T, Szkudelska K (2019). The relevance of AMP-activated protein kinase in insulin-secreting β cells: a potential target for improving β cell function?. J Physiol Biochem.

[92] Ronnebaum SM, Joseph JW, Ilkayeva O (2008). Chronic suppression of acetyl-CoA carboxylase 1 in β-cells impairs insulin secretion via inhibition of glucose rather than lipid metabolism. J Biol Chem.

[93] MacDonald MJ, Dobrzyn A, Ntambi J, Stoker SW (2008). The role of rapid lipogenesis in insulin secretion: insulin secretagogues acutely alter lipid composition of INS-1 832/13 cells. Arch Biochem Biophys.

[94] Yavari A, Stocker CJ, Ghaffari S (2016). Chronic activation of γ2 AMPK induces obesity and reduces β cell function. Cell Metab.

[95] Lin SC, Hardie DG (2018). AMPK: sensing glucose as well as cellular energy status. Cell Metab.

[96] Hinchy EC, Gruszczyk AV, Willows R (2018). Mitochondria-derived ROS activate AMP-activated protein kinase (AMPK) indirectly. J Biol Chem.

[97] Gao T, McKenna B, Li C (2014). Pdx1 maintains β cell identity and function by repressing an α cell program. Cell Metab.

[98] Taylor BL, Liu FF, Sander M (2013). Nkx6.1 is essential for maintaining the functional state of pancreatic Beta cells. Cell Rep.

[99] Gu C, Stein GH, Pan N (2010). Pancreatic beta cells require NeuroD to achieve and maintain functional maturity. Cell Metab.

[100] Kaneto H, Miyatsuka T, Kawamori D (2008). PDX-1 and MafA play a crucial role in pancreatic β-cell differentiation and maintenance of mature β-cell function. Endocr J.

[101] Wortham M, Sander M (2021). Transcriptional mechanisms of pancreatic β-cell maturation and functional adaptation. Trends Endocrinol Metab.

[102] Huang JL, Lee S, Hoek P, van der Meulen T, Van R, Huising MO (2020). Genetic deletion of urocortin 3 does not prevent functional maturation of beta cells. J Endocrinol.

[103] Wortham M, Sander M (2016). Mechanisms of β-cell functional adaptation to changes in workload. Diabetes Obes Metab.

[104] Nishimura W, Takahashi S, Yasuda K (2014). MafA is critical for maintenance of the mature beta cell phenotype in mice. Diabetologia.

[105] Aguayo-Mazzucato C, Zavacki AM, Marinelarena A (2013). Thyroid hormone promotes postnatal rat pancreatic β-cell development and glucose-responsive insulin secretion through MAFA. Diabetes.

[106] Yoshihara E, Wei Z, Lin CS (2016). ERRγ is required for the metabolic maturation of therapeutically functional glucose-responsive β cells. Cell Metab.

[107] Goodyer WR, Gu X, Liu Y, Bottino R, Crabtree GR, Kim SK (2012). Neonatal β cell development in mice and humans is regulated by Calcineurin/NFAT. Dev Cell.

[108] Arda HE, Benitez CM, Kim SK (2013). Gene regulatory networks governing pancreas development. Dev Cell.

[109] Piccand J, Strasser P, Hodson DJ (2014). Rfx6 maintains the functional identity of adult pancreatic β cells. Cell Rep.

[110] Van de Velde S, Wiater E, Tran M, Hwang Y, Cole PA, Montminy M (2019). CREB promotes Beta cell gene expression by targeting its coactivators to tissue-specific enhancers. Mol Cell Biol.

[111] Segerstolpe Å, Palasantza A, Eliasson P (2016). Single-cell transcriptome profiling of human pancreatic islets in health and type 2 diabetes. Cell Metab.

[112] Lawlor N, George J, Bolisetty M (2017). Single-cell transcriptomes identify human islet cell signatures and reveal cell-type-specific expression changes in type 2 diabetes. Genome Res.

[113] Muraro MJ, Dharmadhikari G, Grün D (2016). A single-cell transcriptome atlas of the human pancreas. Cell Syst.

[114] Li J, Klughammer J, Farlik M (2016). Single-cell transcriptomes reveal characteristic features of human pancreatic islet cell types. EMBO Rep.

[115] Xin Y, Kim J, Okamoto H (2016). RNA sequencing of single human islet cells reveals type 2 diabetes genes. Cell Metab.

[116] Dorrell C, Schug J, Canaday PS (2016). Human islets contain four distinct subtypes of β cells. Nat Commun.

[117] Pound LD, Oeser JK, O’Brien TP (2013). G6PC2: a negative regulator of basal glucose-stimulated insulin secretion. Diabetes.

[118] Bosma KJ, Rahim M, Oeser JK, McGuinness OP, Young JD, O’Brien RM (2020). G6PC2 confers protection against hypoglycemia upon ketogenic diet feeding and prolonged fasting. Mol Metab.

[119] Massa L, Baltrusch S, Okar DA, Lange AJ, Lenzen S, Tiedge M (2004). Interaction of 6-Phosphofructo-2-kinase/Fructose-2,6-Bisphosphatase (PFK-2/FBPase-2) with Glucokinase activates glucose phosphorylation and glucose metabolism in insulin-producing cells. Diabetes.

[120] Arden C, Hampson LJ, Huang GC (2008). A role for PFK-2/FBPase-2, as distinct from fructose 2,6-bisphosphate, in regulation of insulin secretion in pancreatic β-cells. Biochem J.

[121] Arda HE, Li L, Tsai J (2016). Age-dependent pancreatic gene regulation reveals mechanisms governing human β cell function. Cell Metab.

[122] Bevacqua RJ, Lam JY, Peiris H (2021). SIX2 and SIX3 coordinately regulate functional maturity and fate of human pancreatic β cells. Genes Dev.

[123] Velazco-Cruz L, Goedegebuure MM, Maxwell KG, Augsornworawat P, Hogrebe NJ, Millman JR (2020). SIX2 regulates human β cell differentiation from stem cells and functional maturation in vitro. Cell Rep.

[124] Siehler J, Blöchinger AK, Meier M, Lickert H (2021). Engineering islets from stem cells for advanced therapies of diabetes. Nat Rev Drug Discov.

[125] Balboa D, Iworima DG, Kieffer TJ (2021). Human pluripotent stem cells to model islet defects in diabetes. Front Endocrinol (Lausanne).

[126] Yoshihara E, O’Connor C, Gasser E (2020). Immune-evasive human islet-like organoids ameliorate diabetes. Nature.

[127] Scheibner K, Bakhti M, Bastidas-Ponce A, Lickert H (2019). Wnt signaling: implications in endoderm development and pancreas organogenesis. Curr Opin Cell Biol.

[128] Sharon N, Vanderhooft J, Straubhaar J (2019). Wnt signaling separates the progenitor and endocrine compartments during pancreas development. Cell Rep.

[129] Yung T, Poon F, Liang M et al (2019) Sufu- and Spop-mediated downregulation of Hedgehog signaling promotes beta cell differentiation through organ-specific niche signals. Nat Commun 10(1). 10.1038/s41467-019-12624-5 10.1038/s41467-019-12624-5 PMC6789033 31604927

[130] Vethe H, Ghila L, Berle M (2019). The effect of WnT pathway modulators on human iPSC-derived pancreatic beta cell maturation. Front Endocrinol (Lausanne).

[131] Nguyen-Tu MS, Da Silva XG, Leclerc I, Rutter GA (2018). Transcription factor-7–like 2 (TCF7L2) gene acts downstream of the Lkb1/Stk11 kinase to control mTOR signaling, cell growth, and insulin secretion. J Biol Chem.

[132] Velazco-Cruz L, Song J, Maxwell KG (2019). Acquisition of Dynamic Function in human stem cell-derived β cells. Stem Cell Rep.

[133] Sakhneny L, Mueller L, Schonblum A (2021). The postnatal pancreatic microenvironment guides β cell maturation through BMP4 production. Dev Cell.

[134] Christensen GL, Jacobsen MLB, Wendt A (2015). Bone morphogenetic protein 4 inhibits insulin secretion from rodent beta cells through regulation of calbindin1 expression and reduced voltage-dependent calcium currents. Diabetologia.

[135] Aguayo-Mazzucato C, Dilenno A, Hollister-Lock J (2015). MAFA and T3 drive maturation of both fetal human islets and insulin-producing cells differentiated from hESC. J Clin Endocrinol Metab.

[136] Syed SK, Kauffman AL, Beavers LS (2013). Ectonucleotidase NTPDase3 is abundant in pancreatic β-cells and regulates glucose-induced insulin secretion. Am J Physiol Endocrinol Metab.

[137] Nica AC, Ongen H, Irminger JC (2013). Cell-type, allelic, and genetic signatures in the human pancreatic beta cell transcriptome. Genome Res.

[138] Bartley C, Brun T, Oberhauser L (2019). Chronic fructose renders pancreatic β-cells hyper-responsive to glucose-stimulated insulin secretion through extracellular ATP signaling. Am J Physiol Metab.

[139] Jacovetti C, Matkovich SJ, Rodriguez-Trejo A, Guay C, Regazzi R (2015) Postnatal β-cell maturation is associated with islet-specific microRNA changes induced by nutrient shifts at weaning. Nat Commun 6(May). 10.1038/ncomms9084 10.1038/ncomms9084 PMC4569696 26330140

[140] Guay C, Regazzi R (2016). New emerging tasks for microRNAs in the control of β-cell activities. Biochim Biophys Acta Mol Cell Biol Lipids.

[141] Sałówka A, Martinez-Sanchez A (2021). Molecular mechanisms of nutrient-mediated regulation of MicroRNAs in pancreatic β-cells. Front Endocrinol (Lausanne).

[142] Pullen TJ, da Silva XG, Kelsey G, Rutter GA (2011). miR-29a and miR-29b contribute to pancreatic β-cell-specific silencing of Monocarboxylate transporter 1 (Mct1). Mol Cell Biol.

[143] Li Y, Deng S, Peng J (2019). MicroRNA-223 is essential for maintaining functional β-cell mass during diabetes through inhibiting both FOXO1 and SOX6 pathways. J Biol Chem.

[144] López-Beas J, Capilla-González V, Aguilera Y (2018). miR-7 modulates hESC differentiation into insulin-producing Beta-like cells and contributes to cell maturation. Mol Ther - Nucleic Acids.

[145] Wang Y, Liu J, Liu C, Naji A, Stoffers DA (2013). MicroRNA-7 regulates the mTOR pathway and proliferation in adult pancreatic β-cells. Diabetes.

[146] Dumortier O, Fabris G, Pisani DF (2020). microRNA-375 regulates glucose metabolism-related signaling for insulin secretion. J Endocrinol.

[147] Sun X, Wang L, Obayomi SMB, Wei Z (2021). Epigenetic regulation of β cell identity and dysfunction. Front Endocrinol (Lausanne).

[148] Parveen N, Dhawan S (2021). DNA methylation patterning and the regulation of Beta cell homeostasis. Front Endocrinol (Lausanne).

[149] Zhou JX, Dhawan S, Fu H (2013). Combined modulation of polycomb and trithorax genes rejuvenates β cell replication. J Clin Invest.

[150] Sharma U, Rando OJ (2017). Metabolic inputs into the epigenome. Cell Metab.

