# Supplementary material for: Maturation of beta cells: lessons from in vivo and in vitro models
Source: Diabetologia. 2022 Mar 4;65(6):917–30. doi: 10.1007/s00125-022-05672-y (PMC9076740; doi:10.1007/s00125-022-05672-y)
Supplement: Supplementary file 1 — (PPTX 332 kb) [file 125_2022_5672_MOESM1_ESM.pptx]

## Slide 1
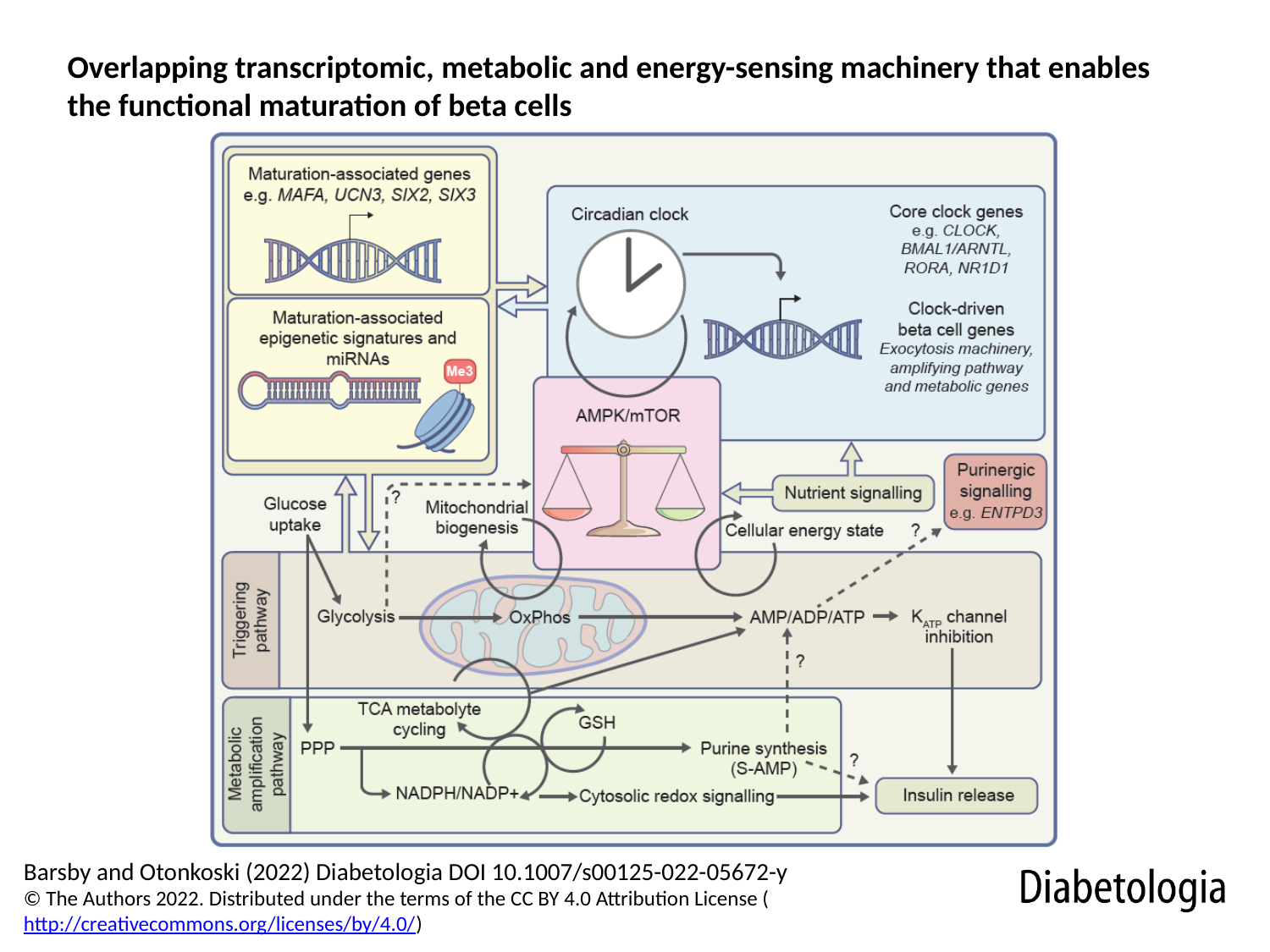

Overlapping transcriptomic, metabolic and energy-sensing machinery that enables the functional maturation of beta cells
Barsby and Otonkoski (2022) Diabetologia DOI 10.1007/s00125-022-05672-y
© The Authors 2022. Distributed under the terms of the CC BY 4.0 Attribution License (http://creativecommons.org/licenses/by/4.0/)

## Slide 2
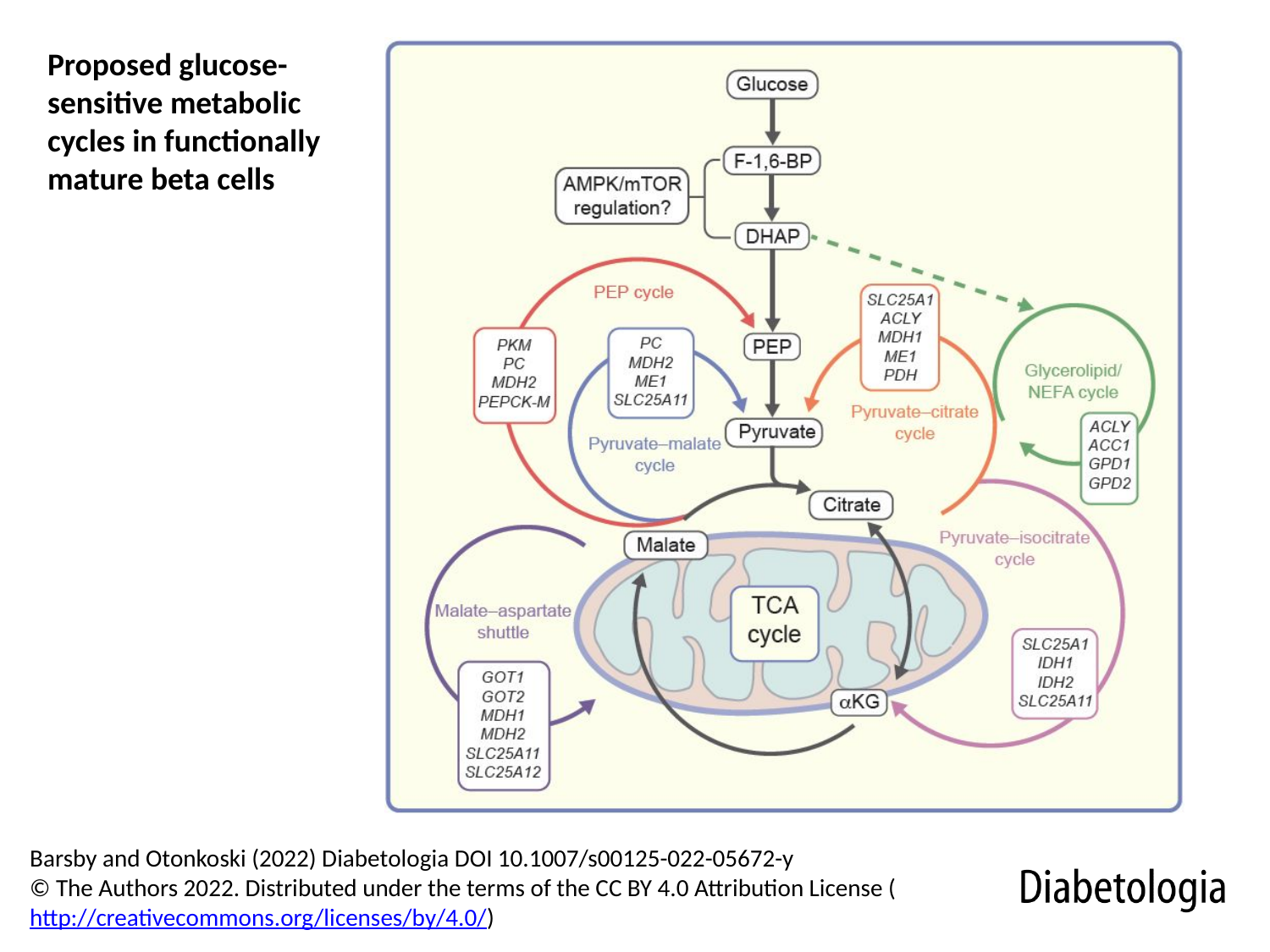

Proposed glucose-sensitive metabolic cycles in functionally mature beta cells
Barsby and Otonkoski (2022) Diabetologia DOI 10.1007/s00125-022-05672-y
© The Authors 2022. Distributed under the terms of the CC BY 4.0 Attribution License (http://creativecommons.org/licenses/by/4.0/)
